# Comparative Analysis of Water-Soluble Polysaccharides from *Dendrobium* Second Love ‘Tokimeki’ and *Dendrobium nobile* in Structure, Antioxidant, and Anti-Tumor Activity In Vitro

**DOI:** 10.3390/ijms241210361

**Published:** 2023-06-20

**Authors:** Guangying Ye, Jinhui Zhang, Xiaoli Xu, Canbiao Zeng, Qingsheng Ye, Zaihua Wang

**Affiliations:** 1 Guangdong Key Lab of Ornamental Plant Germplasm Innovation and Utilization, Institute of Environmental Horticulture, Guangdong Academy of Agricultural Sciences, Guangzhou 510640, China; 2 Instrumental Analysis and Research Center, South China Agricultural University, Guangzhou 510642, China; 3 Guangdong Province Key Lab for Biotechnology of Plant Development, College of Life Science, South China Normal University, Guangzhou 510631, China

**Keywords:** antioxidant activity, anti-tumor activity, polysaccharide, *Dendrobium nobile*, *Dendrobium* Second Love ‘Tokimeki’

## Abstract

With potential anti-tumor and antioxidant properties, the polysaccharide content of *D. nobile* is relatively lower than that of the other medicinal *Dendrobium.* To find high-content polysaccharide resources, the polysaccharide (DHPP-Ⅰs) was prepared from *D.* Second Love ‘Tokimeki’ (a *D. nobile* hybrid) and compared with DNPP-Ⅰs from *D. nobile*. DHPP-Is (Mn 31.09 kDa) and DNPP-Is (Mn 46.65 kDa) were found to be *O*-acetylated glucomannans (-Glc*p*-(1,4) and *O*-acetylated-D-Man*p*-(1,4) backbones), analogous to other *Dendrobium* polysaccharides. DHPP-Ⅰs had higher glucose content (31.1%) and a lower degree (0.16) of acetylation than DNPP-Ⅰs (15.8%, 0.28). Meanwhile, DHPP-Ⅰs and DNPP-Ⅰs had the same ability in the radical scavenging assay, which was milder than the control of Vc. Both DHPP-Is and DNPP-Is inhibited *SPC-A-1* cell proliferation in vitro, with obvious differences in dose concentrations (0.5–2.0 mg/mL) and treatment times (24–72 h). Therefore, the antioxidant activity of DHPP-Ⅰs and DNPP-Ⅰs is not associated with distinction in anti-proliferative activity. As a glucomannan derived from non-medicinal *Dendrobium*, DHPP-Ⅰs has similar bioactivity to other medicinal *Dendrobium,* and this could serve as a starting point for studying the conformational–bioactivity relationship of *Dendrobium* polysaccharides.

## 1. Introduction

Natural polysaccharides are essential biomacromolecules that play a key role in the fundamental biological processes of life [1] and have important and diverse biological activities, such as anti-tumor, immunomodulatory, antioxidant, and anti-diabetic properties [2]. Due to differences in monosaccharide composition and configuration, glycosidic bond and functional group modification, the number of polysaccharide structures is theoretically almost uncountable [3]. However, their functionality, diverse structure, and availability make them an extremely important basis for novel chemical entities with pharmacological activity [4,5]. Therefore, bioactive polysaccharides are increasingly being recognized as potential therapeutic targets in the prevention and treatment of diseases.

Dozens of polysaccharides have been shown to exhibit good anti-tumor activity through DNA damage, cell cycle arrest, and nitric oxide production [6]. Some of these have had milder side effects than conventional chemotherapy drugs [7]. Some polysaccharides have been reported to regulate T cells, B lymphocytes, macrophages, and natural killer cells to regulate the immune system through multiple mechanisms [8,9]. Antioxidant, anti-diabetic, kidney-repairing, and anti-inflammatory effects have also been found in a variety of polysaccharides [2]. In general, the properties of natural polysaccharides, such as their molecular weight, the composition of monosaccharides, types and positions of glycosidic bonds, and substituents, are important structural factors affecting bioactivity [10]. Bioactive natural polysaccharides are derived from mushrooms, herbs, fruits, and marine life, which are widely distributed and rich natural resources [11,12]. Among them, herbs are the most common active polysaccharide resource as screening plants.

*Dendrobium* is an excellent plant polysaccharide resource (typical glucomannan), that has been used for thousands of years as a traditional herb, and it is still used by locals to treat diseases [13]. *D. officinale* polysaccharides have been found to reduce colon tumor formation and growth at an in vivo dose of 100 mg/kg [14]. Polysaccharides from *D. huoshanense* strains resulted in a cell apoptosis rate of 25.05% in MFC cells at concentrations of 100 μg/mL in vivo [15]. Many *Dendrobium* polysaccharides can increase the production of cytokines (*TNF-α*, *IL-2*, *IL-4*, *IL-6*, etc.) and enhance lymphocyte expression to achieve anti-tumor effects [16]. In addition, these polysaccharides have a certain ability to scavenge free radicals, such as ABTS, DPPH, OH, and O^2−^ [17]. Considering all of these factors, the anti-tumor activity of *Dendrobium* polysaccharides is its most important biological activity and the main direction of polysaccharide resource development.

As a traditional herb rich in alkaloids, *D. nobile* is stipulated in Chinese pharmacopeia for medical-related usage [18]. The polysaccharides of *D. nobile*, a typical medicinal *Dendrobium*, exhibit anti-tumor and antioxidant properties [19,20]. Although *D. nobile* normally has a polysaccharide concentration of 10% [21], this is much lower than the polysaccharide contents of other *Dendrobium* species (such as *D. officinale*, *D. chrysotoxum*, *D. huoshanense*, and *D. fimbriatum*) as stipulated in Chinese pharmacopeia [22]. The hybrid progeny of *D. nobile* with the highest polysaccharide content is *D.* Second Love ‘Tokimeki’ [23]. In order to find resources similar to the structure of *D. nobile* with a high polysaccharide content, we analyzed and compared the structural characteristics, antioxidant, and anti-tumor activity of *D.* Second Love ‘Tokimeki’ polysaccharides to those of *D. nobile* polysaccharides. This study aimed determine whether the hybrid cultivar (*D.* Second Love ‘Tokimeki’) could be used as an alternative polysaccharide to *D. nobile*, as well as to reveal similarities among these related polysaccharide compounds that offer a pertinent reference for the development of useful polysaccharide resources.

## 2. Results and Discussion

### 2.1. Isolation and Purification of Polysaccharides

**Figure 1:**
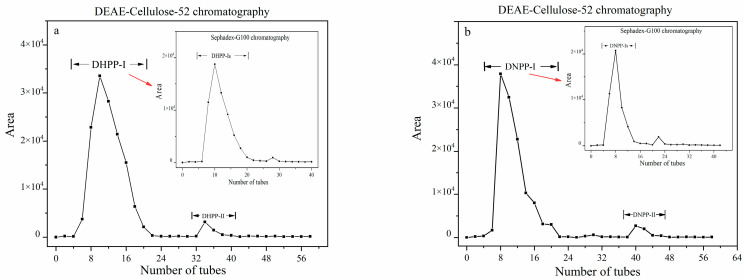
The separation of the polysaccharides from *D.* Second Love ‘Tokimeki’ (DHPP, (**a**)) and *D*. *nobile* (DNPP, (**b**)) by DEAE-Cellulose-52 and Sephadex-G100 column chromatography.

The total polysaccharide content (DW) in the stems of two *Dendrobium* samples was 20.8% (*D.* Second Love ‘Tokimeki’) and 12.0% (*D. nobile*), respectively. Two polysaccharides (2.06 g DHPP and 1.93 g DNPP) were isolated from *D.* Second Love ‘Tokimeki’ and *D. nobile* (100 g dried powder) after extraction and removal of the protein. DHPP and DNPP (100 mg) were sequentially fractionated via anion exchange chromatography and size exclusion chromatography (Figure 1a,b). DHPP-I and DNPP-I were the primary polysaccharides in DHPP and DNPP. For further purification, DHPP-I and DNPP-I were fractionated using Sephadex-G100 with a molecular sieve column to yield DHPP-Is (70.6 mg) and DNPP-Is (65.4 mg). The sugar content of DHPP-Is and DNPP-Is was 98.2% and 98.6% (DW) in the phenol-sulfuric acid assay, and after m-hydroxydiphenyl method testing, the uronic acid content of DHPP-Ⅰs and DNPP-Ⅰs was nearly 5.33% and 3.54% (DW).

### 2.2. Molecular Weight (Mw) Determination and Infrared Spectroscopy

**Figure 2:**
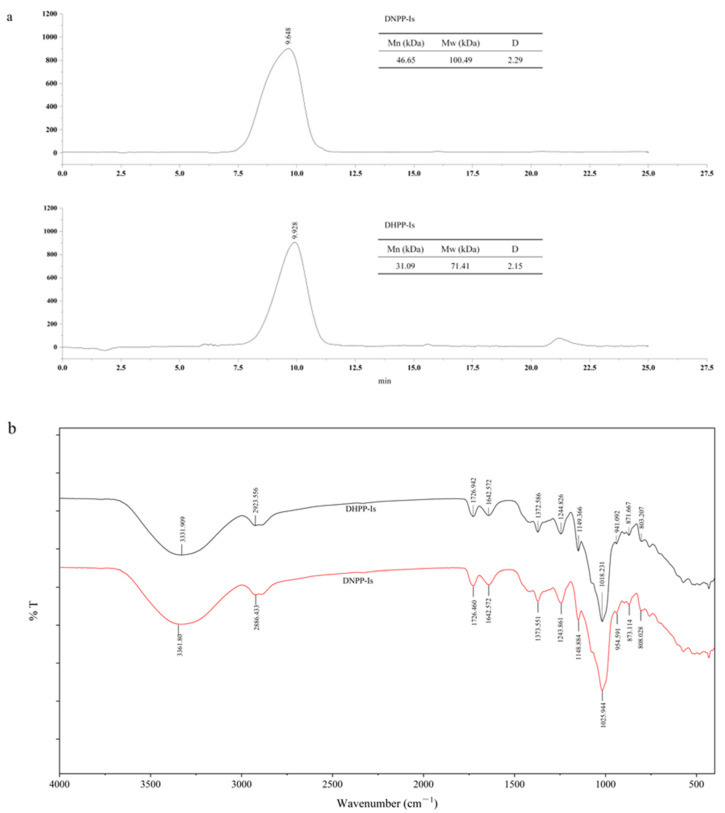
GPC Chromatogram (**a**) and IR spectrum (**b**) of the DHPP−Is and DNPP−Is.

The molecular weights (Mw) of polysaccharides are one of the main factors that determine the configurations and morphologies of the polysaccharides in aqueous solution and affect the microenvironment, which is the principal reflection of their biological activities [24]. The results of GPC showed that the number-average molecular weights (Mn) of DHPP-Ⅰs and DNPP-Ⅰs were estimated to be 31.09 kDa and 46.65 kDa, respectively; the weight-average molecular weights (Mw) of these compounds were 71.41 kDa and 100.49 kDa, respectively (Figure 2a). The Mw of DNPP-Ⅰs was larger than DNPP-Ⅰs.

Infrared (IR) spectroscopy is a useful technique for examining substituent groups of polysaccharides. The typical absorption of the two polysaccharides is shown in Figure 2b. In the X-H stretching region (4000–2500 cm^−1^), there are three absorption peaks, which are the characteristic absorption peaks for polysaccharides. The absorption peaks at 3331 cm^−1^ and 3361 cm^−1^ indicate the absorption of -OH. The two peaks near 3000 cm^−1^ are the C-H stretches of the CH and CH2 groups [25]. Both DHPP-Ⅰs and DNPP-Ⅰs have two absorption peaks of 1726 cm^−1^ and 1642 cm^−1^, which are the carbonyl group (C=O) of acetyl groups and uronic acids [26,27]. The peaks from 1400 cm^−1^ to 1200 cm^−1^ are the bending mode of the C-H in methylene and methyl groups. From 1200–1000 cm^−1^ is a fingerprint region for various polysaccharides, corresponding to ring vibrations overlapped with stretching vibrations of C-O-H side groups and the vibrations of the C-O-C glycoside bands. The absorption peaks of 1149 cm^−1^ and 1148 cm^−1^ correspond to C-O (pyran ring). As the strain vibration of alcohol hydroxide, the peaks of 1061.2 and 1028.1 cm^−1^ mean that pyranose is present in the two polysaccharides. Glucose (871 and 873 cm^−1^) and mannose (808 and 803 cm^−1^) are the typical dominant configurations of the pyranose forms. DHPP-Ⅰs and DNPP-Ⅰs comprise a pyran ring configuration.

### 2.3. Monosaccharide and Methylation Analysis

**Figure 3:**
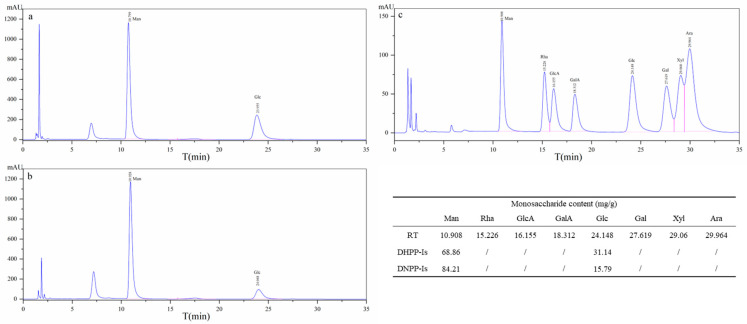
The monosaccharide analysis of DHPP-Is (**a**) and DNPP-Is (**b**), and monosaccharide standard (**c**) by HPLC-UV.

The presence of a C=O group in the IR spectrum suggests that the two polysaccharides may contain uronic acid, which may diminish the methylation effect. After testing with the *m*-hydroxydiphenyl method, the uronic acid content of DHPP-Ⅰs and DNPP-Ⅰs was found to be 5.33% and 3.54% (DW), respectively. The high content of uronic acid in two polysaccharides was reduced before methylation analysis. The structural characteristics of polysaccharides are based on the component monosaccharides. The monosaccharide content of the DHPP-Is and DNPP-Is samples was evaluated by HPLC-UV following hydrolysis and derivatization (PMP) (Figure 3). Referring to the monosaccharide standards (Figure 3c), mannose and glucose are the two major monosaccharides for both polysaccharides. The ratio of mannose to glucose is about 2.5:1 and 5.5:1 in DHPP-Ⅰs and DNPP-Ⅰs, respectively.

Methylation analysis is a classic and powerful process to analyze the linkage pattern. In combination with the database of PMAA, the types and molar ratios of the methyl glycosides were obtained, and the linkage modes were derived from the ion signals of the GC-MS spectra [28]. As shown in Table 1, the main binding types of DHPP-Is and DNPP-Is are 1,4-Man*p* and 1,4-Glc*p*. The proportions of monosaccharides in the two polysaccharides were found to be the same in the monosaccharide composition analysis by HPLC. The backbone likely consists of 1,4-linked glucose and 1,4-linked mannose, consistent with previous studies [29]. The branch may be at C6 of the mannose backbone.

### 2.4. NMR Analysis

**Figure 4:**
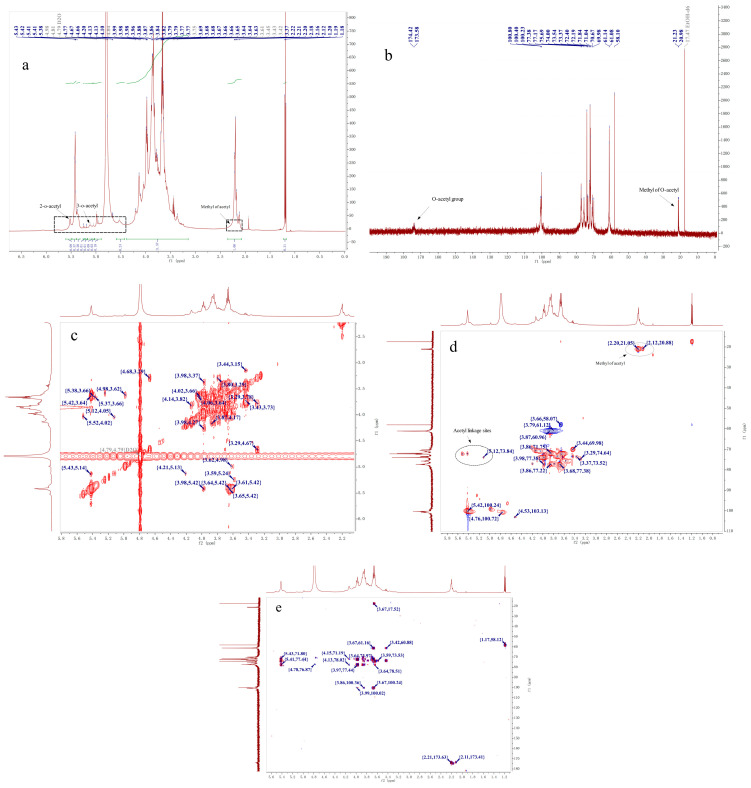
^1^H NMR, ^13^C NMR, and 2DNMR spectra of DHPP-Ⅰs. (**a**): ^1^H NMR spectrum of DHPP-Ⅰs; (**b**): ^13^C NMR spectrum of DHPP-Ⅰs; (**c**): ^1^H-^1^H COSY spectrum of DHPP-Ⅰs; (**d**): HSQC spectrum of DHPP-Ⅰs; (**e**): HMBC spectrum of DHPP-Ⅰs.

**Figure 5:**
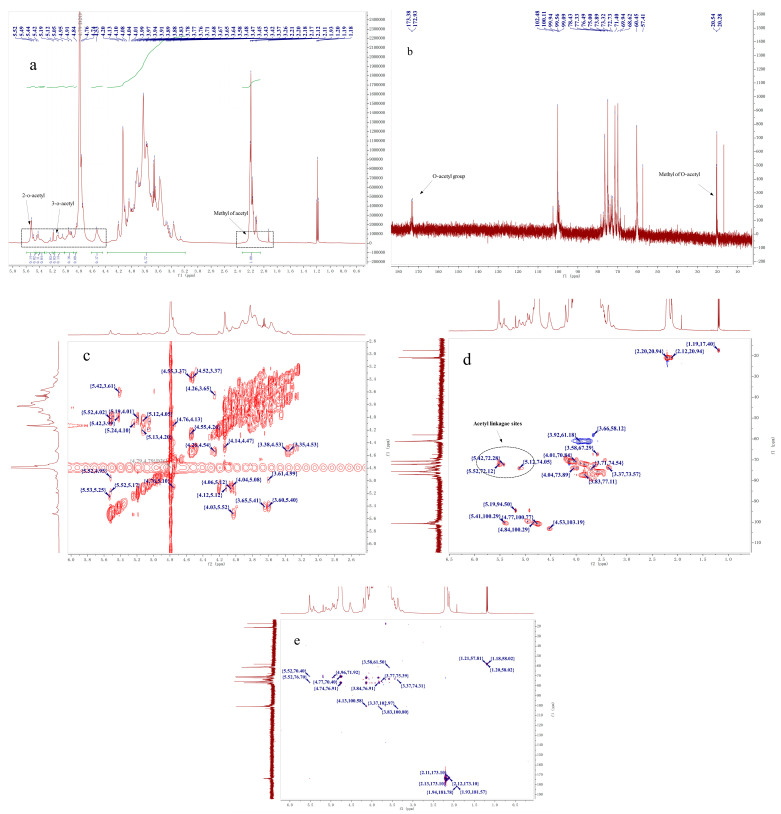
^1^H NMR, ^13^C NMR, and 2DNMR spectra of DNPP-Ⅰs. (**a**): ^1^H NMR spectrum of DNPP-Ⅰs; (**b**): ^13^C NMR spectrum of DNPP-Ⅰs; (**c**): ^1^H-^1^H COSY spectrum of DNPP-Ⅰs; (**d**): HSQC spectrum of DNPP-Ⅰs; (**e**): HMBC spectrum of DNPP-Ⅰs.

**Figure 6:**
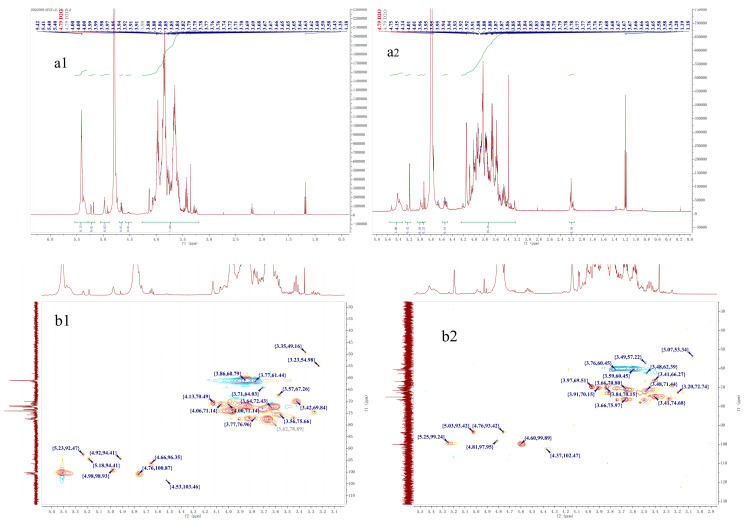
The NMR spectra of acid hydrolysis fragment of DHPP-Ⅰs (**a1**,**b1**) and DNPP-Ⅰs (**a2**,**b2**). (**a1**): ^1^H NMR spectrum; (**b1**): HSQC spectrum; (**a2**): ^1^H NMR spectrum; (**b2**): HSQC spectrum.

The ^1^H, ^13^C NMR, COSY, HMBC, and HSQC spectra of DHPP-Ⅰs (Figure 4) and DNPP-Ⅰs (Figure 5) were used to clarify their structures, which needed to be analyzed separately. The primary structure of the glycan, which included the substituent group, ring protons, and anomeric protons area, was determined by using the ^1^H NMR spectrum [30,31]. In the up-field of ^1^H NMR (Figure 5a and Figure 6a), the signals at ^1^H 2.12 ppm correspond to methyl carbons of an acetyl group, and the hydrogen signal of its corresponding position is evident in the downfield region. At the anomeric region (δ 5.6–4.5 ppm), there are nine major peaks (four peaks < δ 4.8 ppm, five peaks < δ 4.8 ppm), indicating both *β*-configuration and *α*-configuration residues. However, the relative intensities of the group signals in the ^1^H NMR spectrum can provide molar ratios of the monosaccharide residues [32]. Therefore, it is possible to identify that the degrees of *O*-acetylation are 0.16 and 0.28 for a residue in DHPP-Ⅰs and DNPP-Ⅰs, respectively.

In contrast to the ^1^H spectra, ^13^C resonances have substantially more dispersion and a broader spectral range, resulting in much sharper and less overlapping singlet signals. Therefore, the ^13^C NMR spectra was used to determine the anomeric conformation of each residue in DHPP-Is and DNPP-Is (Figure 5b and Figure 6b). The two signals (δ 174.02 and 173.58 ppm) were C=O of acetyl groups in DHPP-Ⅰs and DNPP-Ⅰs, and the CH_3_ of acetyl groups near δ 20.00 ppm. The anomeric carbons region (δ 90–110 ppm), and the ring carbons region (δ 60–80 ppm) were used to determine the primary structure [33]. There were two anomeric carbon signals, primarily at δ 103.36 and 101.10 ppm, indicating that *β*-Glc*p* and *β*-Man*p* are the major components. Compared to DNPP-Ⅰs, DHPP-Ⅰs has more *α*-glucose residues and the same mannose residences. However, due to the difficulty in determining the assignment of each signal in the 60-80 region, two-dimensional NMR analysis was performed.

Based on the COSY, HSQC, and HMBC spectra (Figure 5c–e and Figure 6), the signals are shown in Table 2 and Table 3 for DHPP-Ⅰs and DNPP-Ⅰs, respectively. In the HSQC spectra, the signs in the region of ^1^H downfield to ^13^C upfield were the *O*-acetylated -CH2O and no signs near δ 60 ppm (C6), representing C2 and C3 in the two polysaccharides with 1,4-linkage for the main chain. From the ^1^H-NMR, ^13^C-NMR, and HMBC spectra, the acetyl group can be identified by the signs in ^1^H-NMR (H < 2.4 ppm and H > 5.0 ppm) and ^13^C-NMR (C < 30 ppm and C > 150ppm); HMBC (C > 150 ppm and H < 2.4 ppm) are verified. This clarifies that it is an acetyl group. In the HSQC spectra, the signs in the box (H > 5.0 ppm and C < 90 ppm) were the CH bonds of the acetyl group. Finally, the associated signal from CH connecting acetyl groups in ^1^H-^1^H COZY and HMBC spectra was used to confirm the localization of acetyl esters in the sugar moiety. Since the monosaccharide composition of the two carbohydrates is relatively simple, there was little overlap between the signals of -CH_2_O (from C2 to C5). The detected signals are shown in Table 2 (DHPP-Ⅰs) and Table 3 (DNPP-Ⅰs).

For sample preparation for NMR analysis, the polysaccharides were dissolved in D_2_O and repeatedly lyophilized to remove water and ethanol [34]. However, the o-acetyl signals of CH3 (1.19 and 17.41 ppm) and CH2 (3.66 and 58.38 ppm) were still detected in the NMR spectra [32].

To eliminate the effect of acetyl groups on sugar chains in NMR patterns, DHPP-Ⅰs and DNPP-Ⅰs were hydrolyzed with trifluoroacetic acid to produce low molecular weight fragments (Figure 6). The acetyl groups of the sugar chains were eliminated, according to the HMQC spectrum (Figure 6(b1,b2)). Compared to before hydrolysis (Figure 4d and Figure 5d), the signs of acetyl linkage sites disappeared, indicating that the acid had stripped the acetyl group from the two polysaccharides. However, other signals did not differ significantly between pre- and post-hydrolysis, except for the strength. In the ^1^H NMR spectrum (Figure 6(a1,a2)), the relative integrated area of the heterotopic carbon H (*β*-D-Glc*p*) was reduced compared to that before hydrolysis for DHPP-Ⅰs. In the ^1^H NMR spectra of DNPP-Is, however, the trend was reversed. These results may be due to the increase in the reducing end of the sugar chain after partial hydrolysis, leading to a new equilibrium in the conformation (between α and β in aqueous solution).

**Figure 7:**
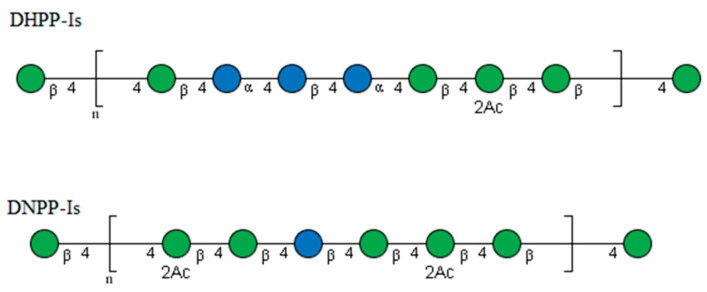
Proposed structures of the repeating units of DHPP-Ⅰs and DNPP-Ⅰs.

Thus, it can be determined that the reducing end of the sugar chain is mannose, while glucose resides primarily within the chain. Combining the above data, the structures of both sugars can be speculated, as shown in Figure 7. They are similar to the reported polysaccharide structure of *Dendrobium officinale* [35]. Compared to DNPP-Ⅰs, DHPP-Ⅰs has a smaller molecular weight, higher *α*-glucose content, and lower acetylation.

### 2.5. Antioxidant Activity

**Figure 8:**
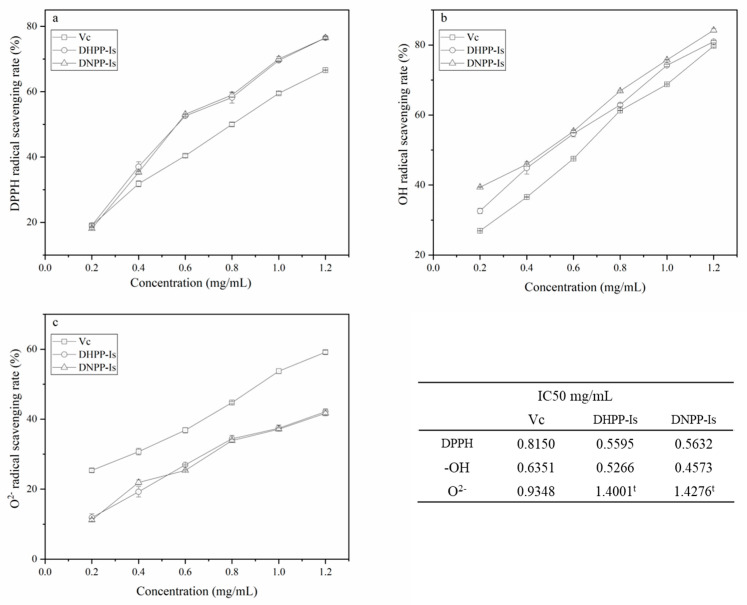
The scavenging rates on DPPH radicals (**a**), hydroxyl radical, (**b**) and superoxide anion radical (**c**) for Vc, DHPP-Ⅰs, and DNPP-Ⅰs (mean ± SD, *n* = 3). ^t^: was the theoretical speculative value.

As one of the biological processes, antioxidant activity has served as the foundation for the various pharmacological activities of polysaccharides [36]. The scavenging rates of DPPH, hydroxyl radical (OH^−^), and superoxide anion radical (O^2−^) of two polysaccharides were investigated (Figure 8). As seen in Figure 8a, the DPPH scavenging rates of DNPP-Ⅰs and DNPP-Ⅰs are extremely similar and are significantly higher than that of Vc at concentrations greater than 0.2 mg/mL. For the OH^−^ radicals (Figure 8b), DNPP-Ⅰs exhibited somewhat greater scavenging rates than DNPP-Ⅰs and Vc. The distances between these three components gradually decreased with the increase in concentration. In addition, it was demonstrated (Figure 8c) that DHPP-Ⅰs and DNPP-Ⅰs have the same scavenging ability on O^2−^, which is lower than that of Vc at the overall concentration level.

DHPP-Is and DNPP-Is performed similarly in the radical scavenger test as refined polysaccharides. The distinctions from Vc were primarily in the various radicals. The radical activity rose in DPPH to O^2−^, whereas the scavenging action of polysaccharides (Vc as a control) decreased. This effect may be caused by multivalent interactions known as the “clustered glycoside” effect [37].

Despite significant differences in molecular weight, monosaccharide content ratio, and degree of acetylation, the antioxidant properties of the two polysaccharides were comparable. This could be a macromolecular action of the polysaccharide after reaching a specific molecular weight, which is unaffected by acetylation and changes in monosaccharide composition. As a result, the biological activity (“clustered glycoside”) generated by the unique macromolecular effect of polysaccharides is still not negligible compared to oligosaccharides and monosaccharides. DNPP-Ⅰs and DHPP-Ⅰs may have an unanticipated effect on a living object.

### 2.6. In Vitro Anti-Proliferative Activity

**Figure 9:**
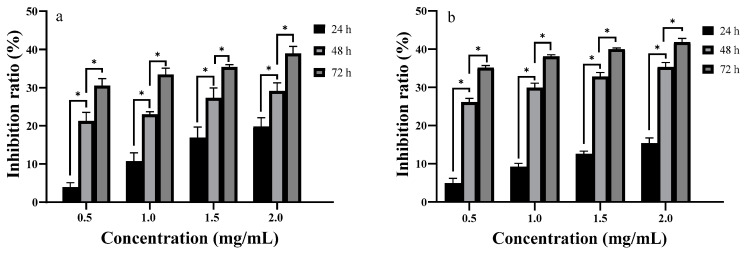
Effects of DHPP-Ⅰs (**a**) and DNPP-Ⅰs (**b**) on the inhibition ratios of *SPC-A-1* in vitro (mean ± SD, n = 3). *: significant differences in different treatment groups within the same times (*p* < 0.01).

Cancer cells that were frequently under abnormal oxidative stress were still able to maintain redox homeostasis [38]. Consequently, inhibiting the normal process of cancer cells by influencing their oxidative stress with antioxidants is a feasible anti-tumor therapy [39]. Since anti-tumor activity is an essential feature of polysaccharide activity [2], we investigated the anti-proliferative activity of DHPP-Ⅰs and DNPP-Ⅰs in vitro. The inhibitory effect of DHPP-Ⅰs and DNPP-Ⅰs (at different doses and incubation times) on the growth of SPC-A-1 cells is shown in Figure 9. Throughout the process, the concentrations of polysaccharides increased from 0.5 mg/mL to 2.0 mg/mL within consecutive processing periods (24-h, 48-h, and 72-h). Both the longer processing time and the higher concentration of DHPP-Ⅰs and DNPP-Ⅰs inhibited SPC-A-1 cell proliferation. There was a dose–response relationship between polysaccharides concentration and inhibition of SPC-A-1 cell proliferation, and the inhibitory impact was cumulative as the treatment period was extended. Meanwhile, DNPP-Ⅰs showed higher anti-proliferation than DHPP-Ⅰs, although both fell short of the 50% threshold.

After a 24 h treatment, the inhibitory ratio of DHPP-Ⅰs rose from 3.96% to 19.80% at concentrations ranging from 0.5 mg/mL to 2.0 mg/mL, outperforming DNPP-Ⅰs. However, at 48 h, the inhibition ratios of DNPP-Is were significantly higher than those of DHPP-Ⅰs. From the unit time suppression effect, the time effects become the main factor affecting the inhibition rate for DHPP-Ⅰs and DNPP-Ⅰs after the 48 h treatment. These results revealed that treatment with DHPP-Ⅰs and DNPP-Ⅰs inhibited SPC-A-1 cell proliferation in a time-dependent manner. Comparing the two polysaccharides, DHPP-Ⅰs had a higher inhibitory impact for a shorter period (24 h) and DNPP-Ⅰs had a more sustained inhibitory effect. However, the similar antioxidant activities of the two polysaccharides may indicate that antioxidant activity may not be the anti-tumor mechanism of polysaccharides. The suppression of tumor proliferation effects of the two polysaccharides may be induced by “clustered glycoside”, while the degree of acetylation may have a modulating influence on the strength of activity [36].

## 3. Discussion

The results suggest that both DHPP-Ⅰs and DNPP-Ⅰs are macromolecules (Mn of 31.09 kDa and 46.65 kDa, respectively) of glucomannan, with a backbone composed of (1→4)-*α*-D-Glc*p*, (1→4)-*β*-D-Glc*p* and (1→4)-*β*-D-Man*p*. However, DHPP-Ⅰs has a lower molecular weight, higher *α*-glucose content, and lower acetylation than DNPP-Ⅰs, which could be attributed to the genes of its source plant. Further research showed that there is no notable difference in the antioxidant and anti-tumor activities between DHPP-Ⅰs and DNPP-Ⅰs, despite apparent differences in structure. For the conformational relationship, the bioactivity of DHPP-Ⅰs and DNPP-Ⅰs was mainly determined by the multivalent interactions (“clustered glycoside”) of the backbone [37], which is a part of both polysaccharide structures.

Many traditional medicinal *Dendrobium*s, whose active substance is a polysaccharide, are still utilized by the locals for a variety of illnesses [40]. These polysaccharides are mostly glucomannans with similar biological activities but varying strengths [15,29,35,41]. These may indicate that the main chain of glucomannan is the structural basis of activity, while acetylation, molecular weight, or the proportion of other, less abundant monosaccharides are responsible for the differences in activity between polysaccharides, such as DHPP-Ⅰs and DNPP-Ⅰs reported here. In addition, the difference in the degree of acetylation of the two sugars did not result in a substantial change in activity, which is in striking contrast to the effect of acetylation on chitosan [42]. Therefore, investigating polysaccharides may require a different approach than examining small molecules, which must be investigated further in terms of conformational relationships.

Finally, the glucomannans derived from *Dendrobium* species, such as DHPP-Ⅰs and DNPP-Ⅰs, may belong to a different class of polysaccharides compared to other sources. This needs to be further investigated in terms of source, structure, characteristics, and biological activity to provide a theoretical basis for the utilization of these types of glucomannans.

## 4. Materials and Methods

### 4.1. Materials and Chemicals

*D.* Second Love ‘Tokimeki’ was introduced from Japan, and *D. nobile* was collected from Baoshan City (Yunnan Province, China). Two specimens were cultivated in the greenhouse of the Institute of Environmental Horticulture, Guangdong Academy of Agricultural Sciences (China), and authenticated by Professor Xu Yechun. The 2-year-old stems were washed, sliced, and deactivated (105 °C, 15 min), and then dried for 48 h at 65 °C. The dry pieces were crushed and filtered (60-mesh) for later use (stored in a desiccator).

Trypsin (250 U/mg) (Shanghai Boao Biotechnology Co., Ltd., Shanghai, China), the dialysis bag (7.0 kDa) (Shanghai Toscience Biotechnology Co., Ltd., Shanghai, China), cellulose-DEAE-52 and Sephadex-G100 (Sigma-Aldrich (Shanghai) Trading Co., Ltd., Shanghai, China), and the monosaccharide and dextran standards (the Institutes for Food and Drug Control, Beijing, China) were purchase from the corresponding companies. DPPH and ABTS were purchased from Sigma-Aldrich. Cell Counting Kit-8 (CCK-8) was purchased from Sigma-Aldrich. Roswell Park Memorial Institute (RPMI) 1640 medium, and fetal bovine serum (FBS) were purchased from ThermoFisher Scientific Co., Ltd. (Waltham, MA, USA). Human lung adenocarcinoma (SPC-A-1) was obtained from Sun Yat-Sen University (Guangzhou, China). Anhydrous ethanol, n-butanol, petroleum ether (bp: 60–90 °C), acetone, trifluoroacetic acid (TFA), and other reagents used in this study were AR grade.

A rotary evaporator (RE-52 AAB; Shanghai Jiapeng Technology Co., Ltd., Shanghai, China), freeze dryer (VIRTIS; SP Co. Ltd., Warminster, PA, USA), centrifuge (5415D; Eppendorf Corporate Co., Ltd., Hamburg Germany), automatic drop-counting collector (SBS-100 CNC; Huxi Analytical Instrument Factory, Shanghai, China), high-performance liquid chromatography (HPLC) system (Agilent 1260; Agilent, Santa Clara, CA, USA), Fourier-transform infrared spectrometer (FTIR) (Nicolet iS5; Thermo Scientific Co., Ltd., USA), nuclear magnetic resonance (NMR) spectrometer (AVANCE Ⅲ HD 600; Bruker, Germany), GC-MS (GCMS-QP2020, SHIMADZU, Japan), cell incubator (HERACELL150i; Thermo, USA), inverted microscope (TM1700; Yuexian, Guangzhou, China), and microplate reader (Multiskan Mk3; Thermo, USA) were used in this study.

### 4.2. Composition and Structure Analysis of Polysaccharide

#### 4.2.1. Extraction and Purification of Polysaccharide

The dried stem powder (20 g) was successively refluxed with 10× petroleum ether and 80% ethanol for 1 h at 80 °C. Deionized water (10 times volume) was added to the defatted residue and the mixture was kept at 80 °C (water bath) for 2 h. The aqueous extract was obtained after centrifugation and filtration and later concentrated to nearly 40 mL. The concentrated solution was changed from water to 80% ethanol and then kept stationary at 4 °C for 24 h. The crude polysaccharide was obtained with further destarching after centrifugation (12,000 rpm, 4 °C), filtration, and lyophilization.

After complete dissolution, the crude polysaccharide was treated with trypsin (250 U/g, 30 °C for 5 h) and then inactivated (90 °C, 15 min). The mixture was then oscillated for 10 min with 3 volumes of Sevag reagent (chloroform: *n*-butanol; 4:1, *v*/*v*). The supernatants were collected after centrifugation (6000 rpm) and placed in a dialysis bag (7.0 kDa) for dialysis (distilled water, 24 h) [43]. Finally, the polysaccharide was obtained from the dialyzed solution using the abovementioned alcohol precipitation method.

The polysaccharide (100 mg) was sequentially purified by column chromatography (26 mm × 50 cm) with DEAE-cellulose and Sephadex-G100 (600 mL distilled water, 1.0 mL/min). Fractions were collected in 10 mL aliquots form each tube and determined by the refractive index detector (RID) method [44]. The collected fractions were concentrated, precipitated, and lyophilized as mentioned above to obtain water-soluble *Dendrobium* polysaccharide (DPP) for further study.

#### 4.2.2. Determination of Purity, Uronic Acid, and Molecular Weight

The purity of polysaccharides was determined using the phenol-sulfuric acid assay [45]. The samples (1.0 mg/mL) and galacturonic acid were mixed with sulfuric acid (0.0125 M sodium tetraborate) in a test tube and incubated at 100 °C (oil bath) for 5 min. After rapid cooling (ice water bath), *m*-hydroxyphenyl (0.15%, in 0.5 g NaOH solution) was added and thoroughly mixed for 20 min. The absorbance value (OD520 nm) was used to calculate the uronic acid content [46]. The standard curve equation was: OD520 = 0.0118 uronic acid content + 0.0034 (R^2^ = 0.9996).

The HPLC system with a RID detector (35 °C) and two TSK SWXL 4000-3000 columns (30 cm × 7.8 mm) in series was used to determine the molecular weight. The mobile phase was 50 mM NaH_2_PO_4_-Na_2_HPO_4_ (pH 6.7, 0.05% NaN_3_) at 0.8 mL/min with 10 μL injection. Dextran standards (1.0, 5.0, 12.0, 25.0, 50.0, 150.0, and 670.0 kDa) and DPP (5 mg/mL, water) were analyzed with the above system. The linear equation (log MW = −1.7705 RT + 18.03; R^2^ = 0.99, where RT is the retention time) was used to calculate the molecular weight using an Agilent GPC analysis module B.01.01 [28].

#### 4.2.3. Determination of Monosaccharide Composition

The polysaccharide solution (1.0 mL, 1–2 mg/mL) and hydrochloric acid (0.5 mL, 3 M) were mixed and hydrolyzed at 110 °C (oil bath) for 1 h in a glass bottle. After being cooled and neutralized (0.5 mL, 3 M NaOH), the hydrolysate (400 μL) was reacted with 400 μL PMP (5-methyl-2-phenyl-1,2-dihydropyrazol-3-one) methanol solution and 400 μL NaOH (0.3 M) at 70 °C (water bath) for 100 min. After neutralization (0.5 mL, 0.3 M HCl), the water solution was washed three times with chloroform (2.0 mL) and centrifuged for future HPLC analysis. Monosaccharide standards were treated as the above-described method for samples, except for the hydrolysis procedure [47].

HPLC system was performed by Agilent 1260 with Agilent Eclipse XDB-C18 (5 μm, 4.6 × 250 mm) and UV detector (250 nm). The mobile phase was consisted of 0.02 M ammonium acetate (pH 5.2) and acetonitrile (80:20, *v*/*v*) at a flow rate of 0.8 mL/min. A 10 uL sample was injected for analysis after filtration (0.22 μm). The compositional ratio of monosaccharides was calculated [48].

#### 4.2.4. Methylation Analysis

Before methylation analysis of the dried polysaccharides (5 mg), the acidic sugars present were reduced with sodium borodeuteride (180 mg/mL) according to a previous report. The reduced polysaccharide was desalted and lyophilized. The intensely drying polysaccharide (1 mg) was dissolved in 1.0 mL dimethyl sulfoxide (DMSO), and then fine sodium hydroxide (20 mg, 1.0 mL DMSO) and methyl iodide (0.3 mL) were added at 4 °C (ice water bath) for 3 h. After desalination, the resulting products were successively hydrolyzed (trifluoroacetic acid), reduced (NaBD_4_), and acetylated (acetic anhydride) [49]. Finally, the derivatives were dissolved in chloroform for GC-MS.

The GC-MS (GCMS-QP2020, SHIMADZU) was equipped with an MS detector (Quadrupole Mass Spectrometer) and EI Ion source. The capillary chromatographic column was an OV-1 capillary column (30 mL × 0.25 mm, 0.2 mm; 180–210 °C at 2 °C/min, then 210–250 °C at 5 °C/min; split ratio 1:1, 0.6 mL/min He). The injector temperature was set at 250 °C, and the MS Quard temperature was set at 200 °C [29]. The data, quantification of the partially methylated alditol acetates (PMAA) https://glygen.ccrc.uga.edu/ccrc/specdb/ms/pmaa/pframe.html (accessed on 12 May 2022) was used to assess glycosidic linkages [50].

#### 4.2.5. IR and NMR Spectroscopy

The dried DPP (2.0 mg) and KBr (200 mg) were mixed, ground, and pressed into pellets, which were scanned (32 times) with an FT-IR spectrometer from 4000 to 400 cm^−1^ (with 4 cm^−1^ resolution). The spectra were processed using Thermo Scientific OMNIC software.

The polysaccharide samples (30 mg) were repeatedly (3 times) dissolved(D_2_O) and lyophilized. The dried samples were then dissolved in 0.6 mL D_2_O for testing. All NMR experiments were performed on a Bruker Avanve III HD 600 M spectrometer and NMR spectra were recorded at 600 MHz at 295.2 K for 1D NMR (^1^H and ^13^C) and 2D NMR (^1^H-^1^HCOSY, HSQC, and HMBC) [51].

Partial Acid Hydrolysis: The polysaccharides were partially degraded using 0.1 M TFA at 100 °C for 1.5 h, and then cooled to room temperature. Later, the solution was added with methanol and dried with nitrogen to remove TFA. After the hydrolysis product was completely dissolved in water, 3 times ethanol was added to induce precipitation by centrifugation. The polysaccharide fragment was freeze-dried and kept for NMR analysis [52].

### 4.3. Bioactivities of Polysaccharide

#### 4.3.1. Antioxidant Activity Test

The polysaccharide solutions were concentrated with distilled water to a stock concentration of 2 mg/mL and then gradient solutions (ranging from 0.2, 0.4, 0.6, 0.8, 1.0 and 1.2 mg/mL) were prepared by dilution. Using vitamin C (Vc) as a reference substance, the antioxidant scavenging activities of polysaccharides were investigated using the method described [28]. The effects of two polysaccharides (DHPP-Is and DNPP-Is) on DPPH radicals, hydroxyl radicals (OH^−^), and superoxide anion radical (O_2_^−^) were tested using the DPPH assay [53], the hydroxyl radical scavenging assay [54], and the pyrogallol autoxidation method [55]. Scavenging cleavage was evaluated and each assay was repeated three times.

#### 4.3.2. Proliferative Inhibition of SPC-A-1 Cells In Vitro

The two polysaccharides were dissolved in 0.01 M phosphate-buffered saline (PBS, pH 7.4). The polysaccharides solution (4 mg/mL) was mixed with cell culture medium to the final concentrations (0.5, 1.0, 1.5, and 2.0 mg/mL). SPC-A-1 cells were cultured into a single-cell suspension and then replicated and seeded on a microplate (96 well plates, 2.5 × 10^4^ cells per well) in a carbon dioxide incubator (37 °C, 5% CO_2_, 8 h). After aspirating off the culture solution, polysaccharides of different concentrations were added to the microplate and incubated (24, 48, and 72 h) with a blank (only PBS) and repeated three times. Finally, anti-tumor activities were evaluated using the CCK-8 assay in vitro as described [29].

### 4.4. Statistical Analysis

All data are expressed as means ± standard deviation. Origin 9.0 was used for the preparation of figures. Statistical analysis was performed using IBM SPSS Statistics 19.0 software, and Duncan’s multiple comparison method was used to test for significant differences (*p* < 0.05).

## 5. Conclusions

Two polysaccharides (DHPP-Ⅰs and DNPP-Ⅰs) were produced from different *Dendrobium* plants. Structurally, DHPP-Ⅰs and DNPP-Ⅰs belong to the same type of glucomannan (such as other *Dendrobium* polysaccharides, DOP-1-1 etc.) with a similar molecular weight and backbone. DHPP-Ⅰs showed higher glucose content and a lower degree of acetylation than DNPP-Ⅰs. Both DHPP-Ⅰs and DNPP-Ⅰs performed equally well in the scavenger test, which was milder than Vc. Both DHPP-Ⅰs and DNPP-Ⅰs inhibited the proliferation of SPC-A-1 cells in vitro, which differed significantly in dose and treatment duration. As a glucomannan derived from non-medicinal *Dendrobium*, DHPP-Is has similar bioactivity to other medicinal *Dendrobium* species, which provides a reference for the excavation of this type of glucomannan as a new resource. This different *Dendrobium* polysaccharide resource can provide the material basis for further investigation of its conformation–bioactivity relationship.

## Figures and Tables

**Table 1:** Methylation analysis of DHPP-Is and DNPP-Is.

Retention Time (min)	Mass Fraction (m/z)	Linkage Pattern	Molar Ratio	
			DHPP-Is	DNPP-Is
11.967	59, 71, 87, 102, 113, 118, 129, 145, 161, 162, 205	T-Man*p*	2.40	1.82
14.271	59, 71, 87, 99, 102, 113, 118, 129, 131, 162, 233	1,4-Linked Man*p*	63.32	82.67
14.421	59, 71, 87, 99, 102, 113, 118, 129, 131, 162, 233	1,4-Linked Glc*p*	35.99	15.34
16.200	59, 71, 85, 99, 102, 118, 127, 142, 162, 201, 261	1,4,6-Linked Man*p*	0.28	0.15

**Table 2:** ^13^C and ^1^H NMR chemical shifts (ppm) for DHPP-Is.

Sugar Residues	Ratio		1	2	3	4	5	6
→4)-*β*-D-Glc*p*-(1→	2.4	δH	4.53	3.36	3.61	3.71	3.65	-
		δC	103.48	73.56	75.58	79.47	75.93	-
→4)-*α*-D-Glc*p*-(1→	5.2	δH	5.42	3.65	3.98	3.86	3.68	3.78
		δC	100.28	72.19	73.98	71.83	77.36	61.14
→4)-*β*-D-Man*p*-(1→	8.2	δH	4.76	4.08	3.96	3.65	3.99	3.87, 3.84
		δC	100.80	70.78	71.74	74.02	70.38	61.08
→4)-2-*O*-acetyl-*β*-D-Man*p*-(1→	1.5	δH	4.98	5.51	4.02	3.98	3.67	-
		δC	99.28	72.19	74.00	71.84	77.33	-
→4)-3-*O*-acetyl-*β*-D-Man*p*-(1→	1.0	δH	5.41	4.21	5.12	4.05	3.62	-
		δC	100.23	69.27	73.94	70.72	75.52	-

**Table 3:** ^13^C and ^1^H NMR chemical shifts (ppm) for DNPP-Is.

Sugar Residues	Ratio		1	2	3	4	5	6
→4)-*β*-D-Glc*p*-(1→	1.2	δH	4.53	3.36	3.71	4.27	3.65	3.83, 4.00
		δC	103.13	70.63	72.37	74.53	72.37	61.09
→4)-*α*-D-Glc*p*-(1→	0.8	δH	5.42	3.65	3.98	3.86	3.68	3.78
		δC	100.38	72.19	73.98	71.83	77.36	61.14
→4)-*β*-D-Man*p*-(1→	3.6	δH	4.76	3.57	3.83	4.14	3.83	3.92, 3.77
		δC	100.75	75.64	72.05	70.59	77.13	61.09
→4)-2-*O*-acetyl-*β*-D-Man*p*-(1→	1.6	δH	4.96	5.52	4.03	3.82	3.83	-
		δC	99.86	72.24	73.84	72.34	77.09	61.09
→4)-3-*O*-acetyl-*β*-D-Man*p*-(1→	0.8	δH	5.41	4.21	5.12	4.04	3.82	-
		δC	100.41	69.27	73.94	70.72	75.52	61.09
→4)-2,3-*O*-acetyl-*β*-D-Man*p*-(1→	0.2	δH	5.42	5.53	5.24	4.10	3.77	3.94
		δC	100.61	70.44	72.31	69.26	75.61	61.09

## Data Availability

No data was used for the research described in the article.
